# Within-host evolution of bovine *Staphylococcus aureus* selects for a SigB-deficient pathotype characterized by reduced virulence but enhanced proteolytic activity and biofilm formation

**DOI:** 10.1038/s41598-019-49981-6

**Published:** 2019-09-17

**Authors:** Helene Marbach, Katharina Mayer, Claus Vogl, Jean Y. H. Lee, Ian R. Monk, Daniel O. Sordelli, Fernanda R. Buzzola, Monika Ehling-Schulz, Tom Grunert

**Affiliations:** 1 Functional Microbiology, Institute of Microbiology, Department of Pathobiology, University of Veterinary Medicine, Vienna, Austria; 2 Molecular Genetics, Institute of Animal Breeding and Genetics, Department of Biomedical Sciences, University of Veterinary Medicine, Vienna, Austria; 3 Department of Microbiology and Immunology, Peter Doherty Institute for Infection and Immunity, University of Melbourne, Melbourne, Australia; 4 Instituto de Investigaciones en Microbiología y Parasitología Médica (IMPaM), Universidad de Buenos Aires and CONICET, Buenos Aires, Argentina

**Keywords:** Pathogens, Bacterial infection, Clinical microbiology

## Abstract

*Staphylococcus aureus* is a major cause of bovine mastitis, commonly leading to long-lasting, persistent and recurrent infections. Thereby, *S*. *aureus* constantly refines and permanently adapts to the bovine udder environment. In this work, we followed *S*. *aureus* within-host adaptation over the course of three months in a naturally infected dairy cattle with chronic, subclinical mastitis. Whole genome sequence analysis revealed a complete replacement of the initial predominant variant by another isogenic variant. We report for the first time within-host evolution towards a sigma factor SigB-deficient pathotype in *S*. *aureus* bovine mastitis, associated with a single nucleotide polymorphism in *rsbU* (G368A → G122D), a contributor to SigB-functionality. The emerged SigB-deficient pathotype exhibits a substantial shift to new phenotypic traits comprising strong proteolytic activity and poly-*N*-acetylglucosamine (PNAG)-based biofilm production. This possibly unlocks new nutritional resources and promotes immune evasion, presumably facilitating extracellular persistence within the host. Moreover, we observed an adaptation towards attenuated virulence using a mouse infection model. This study extends the role of sigma factor SigB in *S*. *aureus* pathogenesis, so far described to be required for intracellular persistence during chronic infections. Our findings suggest that *S*. *aureus* SigB-deficiency is an alternative mechanism for persistence and underpin the clinical relevance of staphylococcal SigB-deficient variants which are consistently isolated during human chronic infections.

## Introduction

*Staphylococcus aureus* is an important opportunistic bacterial pathogen, infecting humans and a wide range of animals, in particular dairy cattle. Worldwide, *S*. *aureus*-induced bovine mastitis is one of the biggest problems in the dairy industry with strong negative consequences for animal welfare, food safety and productivity^1^. Moreover, cows were recently identified as the main animal source of novel human epidemic clones, underscoring their zoonotic potential^2^. Most of these infections are chronic, subclinical and characterized by long-term intramammary persistence^3^. Current treatment with antibiotics is inefficient which often leads to recurrent infections and finally to premature culling and replacement of incurable cows. Vaccination of cattle against *S*. *aureus* infections has not yet been proven to be effective^4^.

In cattle, two clonal complexes (CC97, CC151) have been described as the most successful lineages which carry distinct molecular genetic features, optimized to induce and maintain infections within the udder microenvironment^5,6^. Several studies reported a recurrent recovery of isolates belonging to the same clone, indicating that *S*. *aureus* host adaptation to the bovine mammary gland results in single or a few persisting subtypes in a herd^7,8^. Niche-specific alterations as a result of within-host adaptation have been associated with a switch to small colony variants (SCVs), concurrent with slow growth and diminished metabolism^9^. In addition, loss of capsular polysaccharide (CP), an important surface-associated virulence factor, is linked to chronic mastitis^10^. Indeed, several studies showed a high prevalence of non-encapsulated *S*. *aureus* strains in bovine, chronic mastitis (up to 86%)^11,12^. As the prevalence of non-encapsulated strains is also higher in chronic than in acute infections in humans^13^, diminished CP expression might be a key *S*. *aureus* phenotypic feature associated with chronicity. Non-encapsulated strains are better equipped to enter epithelial cells, avoiding further immune clearance, whereas CP expressing *S*. *aureus* strains were shown to protect themselves from professional and non-professional phagocytosis^10^. We recently demonstrated that capsule loss, as well as the capacity to invade endothelial cells and to form biofilms are properties linked to long-term persistence in the mammary gland^8^. Nevertheless, the genetic and molecular mechanisms allowing predominant *S*. *aureus* subtypes to successfully persist even for years inside the bovine udder are far from understood.

Recent studies focused on the comparison of genetic and phenotypic traits based on mastitis reference strains, or clinical mastitis isolates of different clonal origin or of different within-herd prevalence^8,14,15^. So far, no study followed *S*. *aureus* within-host adaptation during the progression in chronic, bovine mastitis. In the present study, we analyzed a set of isolates, collected over the course of three months from a cow with chronic, subclinical and untreated bovine mastitis. To obtain a comprehensive picture of within-host adaptation, we carried out an in–depth investigation of the evolution of this pathogen within the bovine host.

## Results

### Following *S*. *aureus* within-host adaptation in the bovine mammary gland

**Figure 1:**
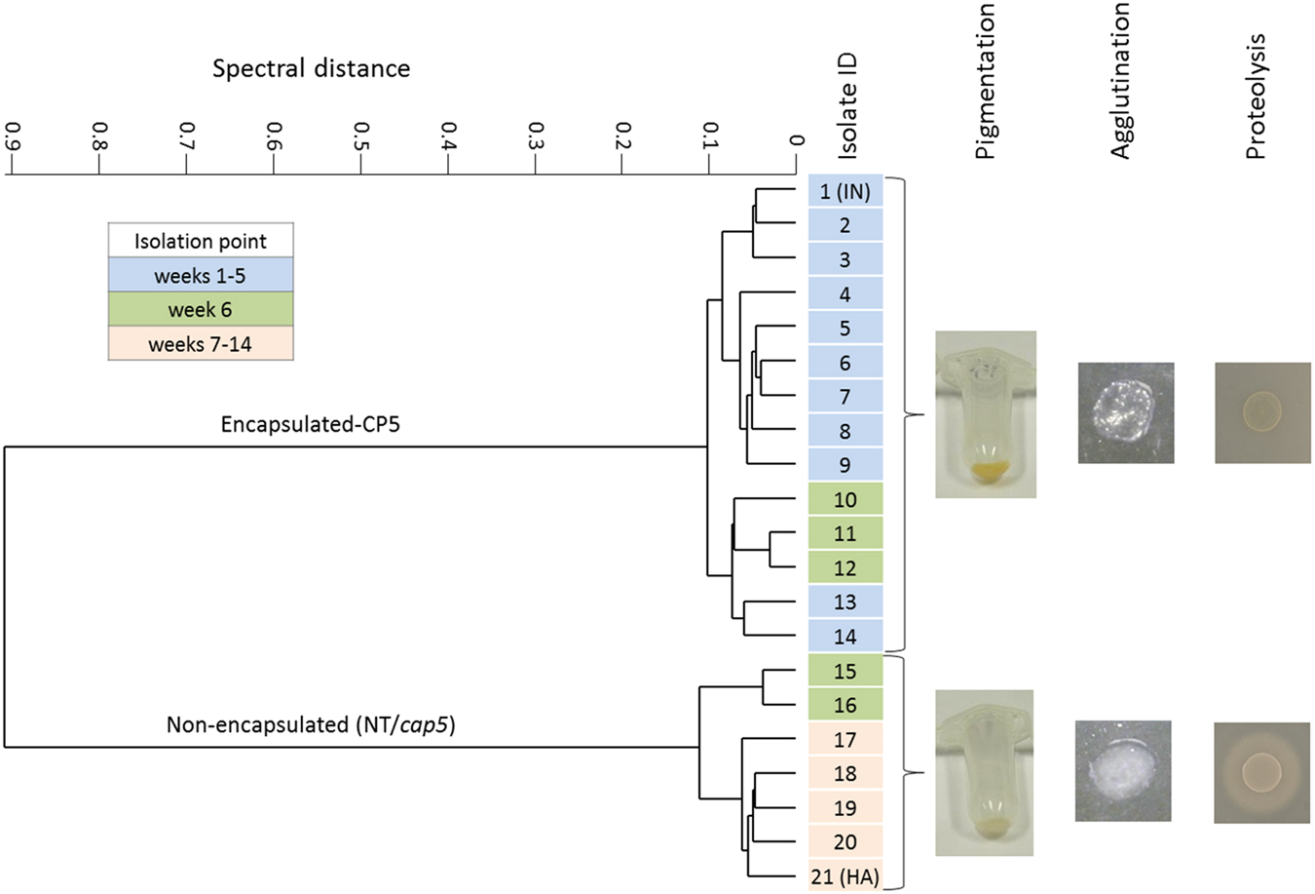
Hierarchical cluster analysis of FTIR spectral data and phenotypic changes. FTIR spectroscopy was used to follow *S*. *aureus* capsular expression of 21 clinical mastitis isolates during the course of a chronic, subclinical bovine mastitis and revealed a transition from encapsulation to non-encapsulation accompanied with changes in distinct phenotypic features. IN, initial isolate; HA, host-adapted isolate.

Since reduced CP expression is an indicator for persistence, we monitored these changes in *S*. *aureus* isolates collected from several dairy herds using a high-throughput capsule serotyping system^16^. From one of the naturally infected dairy cows, 21 longitudinal collected isolates of the same udder quarter depicted the transition from encapsulated to non-encapsulated isolates. Hierarchical cluster analysis of spectral Fourier-transform infrared (FTIR) spectroscopy data (Fig. 1) showed that, up to week five, only encapsulated (CP5) isolates were recovered. At week six, both encapsulated (CP5) and non-encapsulated isolates were detected in the same sample, with only non-encapsulated isolates remaining after week six until the end of the sampling process (week 14). Furthermore, the non-encapsulated isolates showed a loss in pigmentation (Fig. 1 and Fig. S1A), a loss in coagulase activity against rabbit plasma (Fig. 1 and Fig. S1B,C) and strong increased proteolytic activity against casein in milk (Figs 1 and S3A,B).

### Replacement of the initial predominant variant by another isogenic variant, associated with reduced SigB-functionality

**Figure 2:**
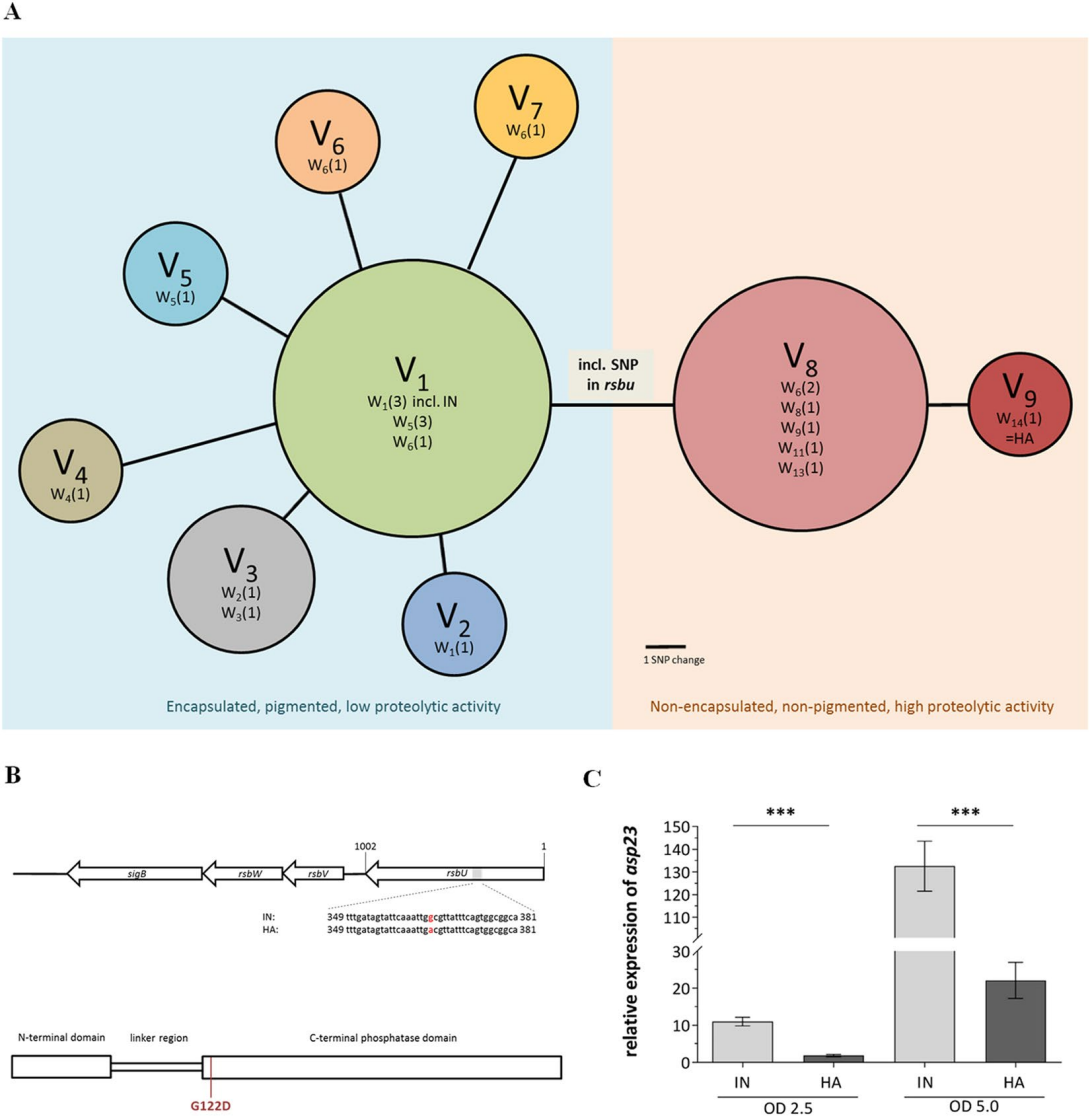
Genomic comparative analysis of 21 collected mastitis isolates, allocation of the RsbU mutation and *asp23* RT-qPCR. (**A**) Each circle represents one isogenic variant (Variant 1 = V_1_ etc.), based on the strains SNP difference, where the size of the circle relates to the number of isolates carrying it and the distance of the circles to each other corresponds to the number of SNP(s). The number of isolates collected at a specific week (W1–14) are shown in parentheses (one isolate at the 1st week, W_1_(1) etc.). See Table S1 for details. v, variants; w, week of sampling; IN, initial isolate; HA, host-adapted isolate. (**B**) Genetic loci encoding for SigB expression in *S*. *aureus*. The grey area in the *rsbU* gene represents the gene sequence in which the SNP (G368A) of the HA isolate was identified. The corresponding protein-missense mutation (G122D) is indicated in red in the RsbU cartoon. (**C**) *Asp23* expression of strains grown in TSB to an OD 600 nm of 2.5 and 5.0 was evaluated by RT-qPCR. The data shown are the means of three independently grown strains and their technical duplicates. Statistical difference between the expression of the initial and host-adapted isolate was determined with unpaired Students *t* test on log10 transformed data. (***P < 0.001). IN, initial isolate; HA, host-adapted isolate.

For in-depth investigation of genetic differences between the isolates, whole-genome sequencing was carried out for all 21 isolates using Illuminas’ MiSeq platform. First, all isolates were assigned to clonal complex CC97, sequence type ST352. Genomic comparative analysis proved clonal ancestry, detecting 17 SNPs amongst the 21 isolates in total, resulting in nine isogenic variants (V_1_ to V_9_) (Fig. 2A). All detected mutations are summarized in Table S1. At the beginning of sampling, the variant V_1_, including the very first isolate (IN), was dominant. Up to week six, further variants derived from V_1_. These isolates were separated from V_1_ by one to four SNPs. From week six onwards, variant V_8_ was observed, which became dominant by week 13. The mutations in the early isolates (V_2_-V_7_) may have incurred a fitness cost, since none of these variants were found at later samplings. By contrast, one of the three single nucleotide mutations, differentiating the isolates of V_8_ from V_1_, might have conferred an advantage, because these isolates quickly replaced V_1_ as the dominant variant. All of the three single nucleotide mutations resulted in an amino acid change: one in the positive regulator of sigma factor SigB, *rsbU* (SA1872), and two in the hypothetical proteins SA0212 and SA0192. The missense mutation identified in *rsbU* (G368A) encodes a glycine/aspartate substitution (G122D) within the C-terminal phosphatase domain (Fig. 2B). In the final week of sampling (week 14), the last isolate collected, which was considered the host-adapted (HA) isolate, harboured an additional SNP in a putative protein (SA1828), although showing the same phenotype as V_8_.

Since we identified one SNP in *rsbU* along with a loss of pigmentation in the HA isolate, we sought to confirm whether the HA isolate is associated with a SigB*-*deficient phenotype assessing the *asp23* gene expression, a marker for SigB-functionality^17^, by qRT-PCR. *Asp23* mRNA levels were significantly lower in HA compared with those in the IN isolate (Fig. 2C). This is consistent with a 73.4-fold (P = 0.013) decrease of the Asp23 protein abundance in HA, obtained from a proteomics approach of whole cellular fraction (data not shown). As controls for SigB deficiency, we included an isogenic strain set, a fully functional SigB (SH1000, a *rsbU* repaired 8325-4 strain) and two strains with known impaired SigB functionality (8325-4, natural 11-bp *rsbU* deletion and SH1000*∆sigB* mutant) and assessed their *asp23* transcription. As expected, the highest gene expression was obtained for the SigB-functional strain SH1000, whereas a significant reduction in *asp23* expression was found for 8325-4 and the SH1000∆sigB under our experimental settings (Fig. S2). Detailed phenotypic characterization of the initial (IN, collected at the first sampling point) and the final isolate (HA, isolated three months later) were conducted.

### Within-host adaptation towards pronounced proteolytic activity

One of the new striking phenotypic features of the host-adapted, SigB-deficient isolates was that they showed a particular strong proteolysis in 1% Skim milk broth as well as on 1% Skim milk agar (Figs 1 and S3). To relatively assess this proteolytic activity, *S*. *aureus* strain 6850 and the isogenic *sigB*-deficient mutant (6850∆*sigB*) were included, where the latter also degraded milk proteins, but to a much lesser extent compared to the HA isolate. No milk protein degradation at all was observed for the IN isolate and the 6850 wild-type strain (Fig. S3A,B).

**Figure 3:**
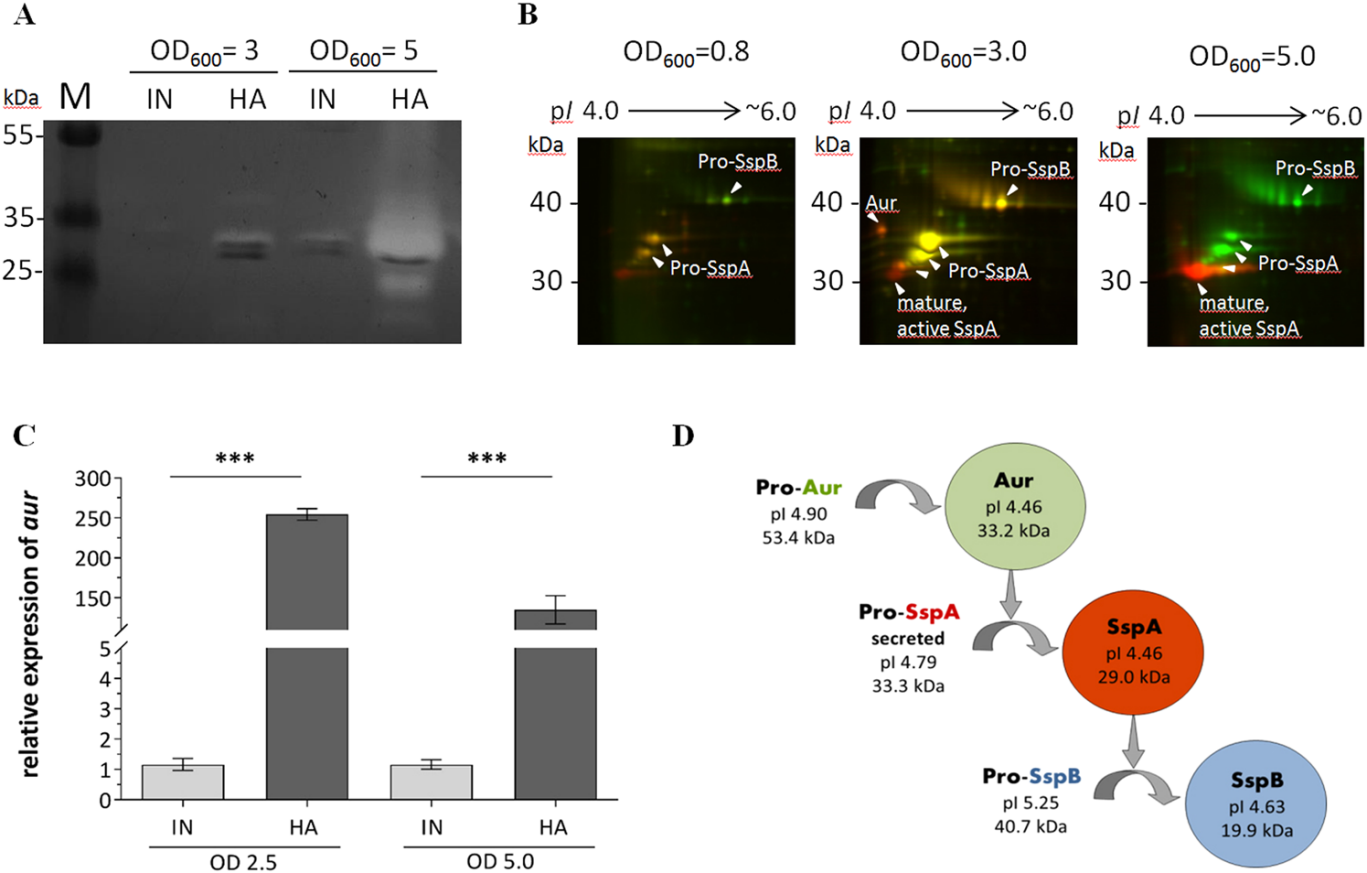
Proteolytic activity. (**A**) Casein gel zymography. SDS-PAGE supplemented with 1% casein as a substrate, where a white band is representative for casein digestion. (**B**) Representative dual-channel gel images of the secretomes of initial (green channel) and host-adapted (red channel) isolate at different OD readings. Differentially abundant proteins were identified by MALDI-TOF-MS/MS (see Fig. S4) (**C**) *aur* expression of strains grown in TSB to an optical density of 2.5 and 5.0. The data shown are the means of three independently grown strains and their technical duplicates. Statistical difference between the expression of the initial and host-adapted isolate was determined with unpaired Students *t* test. (***P < 0.001) (**D**) Proteolytic cascade cartoon, including the proteins’ molecular weight (MW) and isoelectric point (p*I*), adapted from Shaw *et al*.^33^. Original, full length zymogel/ 2D-DIGE gels are shown in the Supplementary Information Fig. S7. IN, initial isolate; HA, host-adapted isolate; SspA, glutamyl endopeptidase; SspB, staphopain B; Aur, zinc metalloprotease aureolysin.

To explore secreted, enzymatically active proteins against casein, the main protein component in milk, zymography was performed using casein as substrate (Fig. 3A). The HA but not the IN isolate completely degraded casein at approximately 30 kDa at an OD_600_ of 5.0. Several subforms of the protein glutamyl endopeptidase (SspA) could be detected by 2D-Differential gel electrophoresis (DIGE) - MALDI-TOF-MS/MS which slightly differ in their isoelectric point (p*I*) and molecular weight (Fig. 3B). In particular, peptide segments from the propeptide of SspA (pro-SspA) were detected in the spots 3, 4 and 5 by MALDI-TOF-MS/MS, but were absent in spot 6 (Fig. S4), which were shown to be released during the step-wise proteolytic processing of SspA activation^18^, more detailed described in Fig. S5. Moreover, zinc metalloprotease aureolysin (Aur) was only detected for the HA, but not in the IN isolates’ supernatant at protein level (Fig. 3B). This was supported by testing the *aur* mRNA expression, by qRT-PCR, which showed a very strong upregulation in the HA compared to the IN isolate (Fig. 3C).

### Within-host adaptation exhibits changes in haemolytic activity

Next, we tested for increased production of alpha hemolysin (Hla), a further well known phenotype of SigB-deficiency^19^. Transcriptional expression of *hla* and secretion of the protein (Hla) revealed a significant increase for the HA compared to the IN isolate (Fig. S6A,B). However, testing for differences in haemolysis by spotting bacteria onto sheep-blood agar (CBA) revealed a decreased haemolytic activity in HA compared to IN (Fig. S6C–E).

### Within-host adaptation reveals reduced cellular uptake, but no differences in intracellular persistence

**Figure 4:**
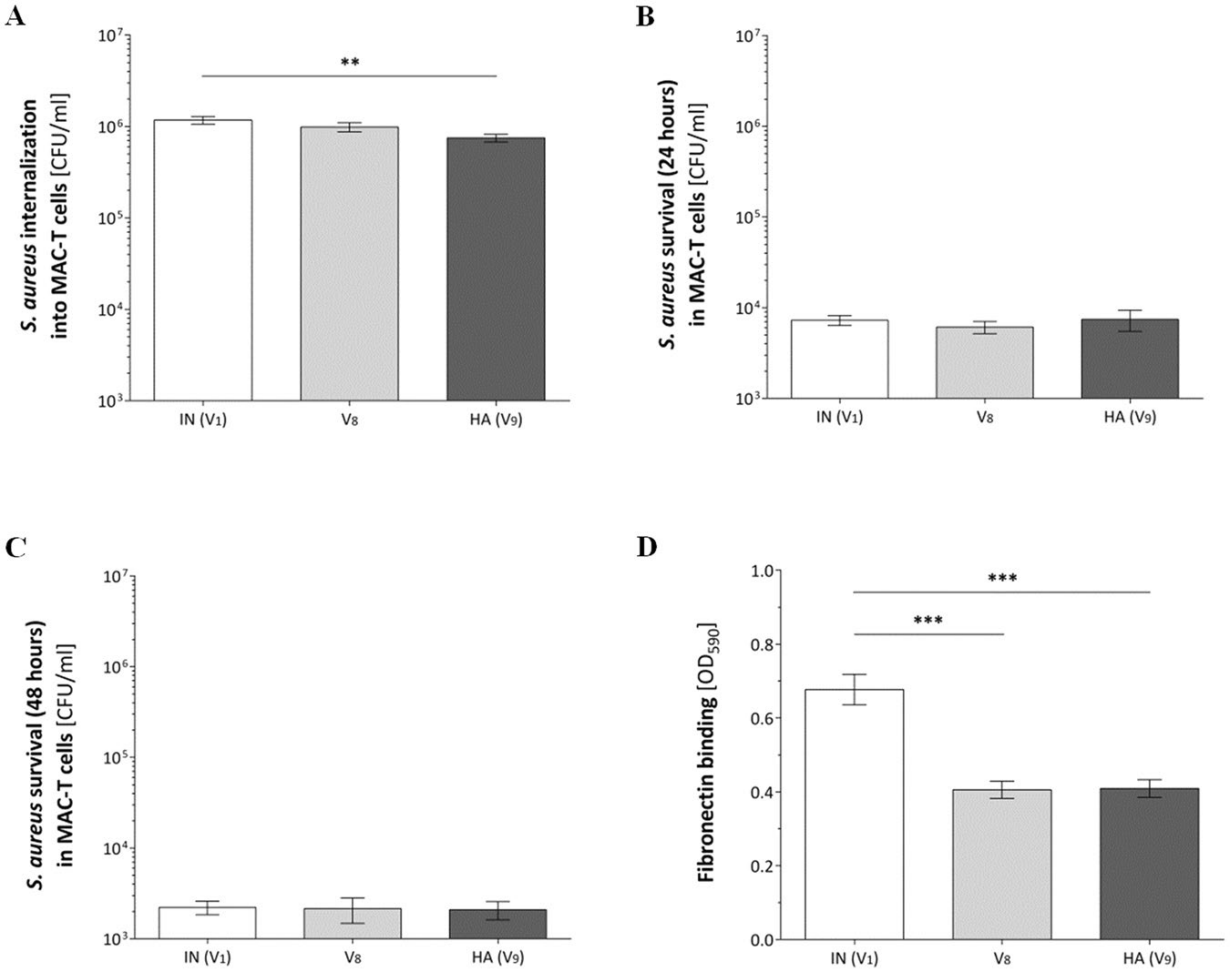
Bacterial internalization and survival in MAC-T cells after 24 hours and 48 hours and binding to immobilized bovine fibronectin. *S*. *aureus* strains were co-cultured with cells of an epithelial bovine cell-line for either 2 (**A**), 24 (**B**) or 48 hours (**C**) post-infection and extracellular bacteria killed by addition of lysostaphin. The number of bacteria internalized and intracellular survival are shown in graphs A-C and represent the mean colony forming units per ml ± SEM, recovered after lysostaphin treatment. Each bar represents a set of three independent experiments, performed at least in triplicate. An unpaired Students *t* test was used to compare between two strains. (**D**) Immobilized fibronectin binding of exponentially phase grown bacteria is shown as the mean ± SEM of three independent experiments, each performed as 6 technical repeats; two-tailed non parametric Mann-Whitney U test comparing the difference in binding of two strains. (**A**–**D**) **P < 0.01; ***P < 0.001). IN, initial isolate; HA, host-adapted isolate; V_8_, the first isolate collected showing a SigB-deficient variant (isolate 15 after week 6).

Bacterial internalization was assessed after a two-hour co-incubation of bacteria and bovine mammary epithelial cells (MAC-T) comparing IN to HA and additionally including the first isolate collected showing a SigB-deficient variant (V_8_; isolate 15 after week 6) to test for phenotypic changes potentially induced by one additional SNP in the HA isolate. Uptake of the HA isolate was significantly lower compared to the IN isolate (P = 0.0042) (Fig. 4A). Subsequent survival experiments in MAC-T cells were conducted at 24- and 48-hours post-infection. No difference in intracellular persistence in MAC-T cells were observed at any of the two time points for the IN and HA isolate (Fig. 4B,C). In addition to its lower internalization, the HA isolate also exhibited a significantly decreased binding to immobilized bovine fibronectin (IN; P < 0.001), identified by a standard crystal violet protein binding assay (Fig. 4D). We obtained no differences neither in internalization, persistence nor binding to fibronectin between both SigB-deficient variants, V_8_ and V_9_ (HA).

### Within-host adaptation promotes PNAG-based biofilm production

**Figure 5:**
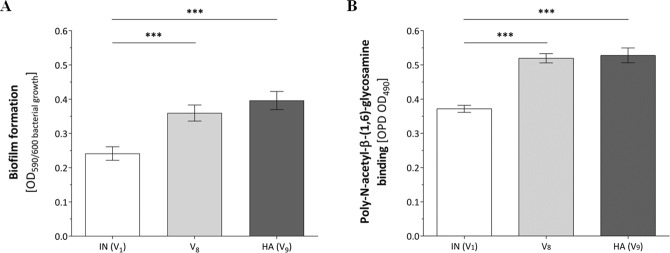
Biofilm formation and PNAG production. Biofilm formation and PNAG production by statically grown bacteria, in TSB for 24 hours at 37 °C. (**A**) Data shown represent the normalized crystal violet - biofilm results (OD 590 nm) divided by the individual bacterial growth identified at an optical density of 600 nm. (**B**) Data shown are the results of bacterial PNAG bound to wheat germ agglutinin horseradish peroxidase conjugate (OD 490 nm). (**A**,**B**) Both graphs show the results as the mean ± SEM of three independent experiments, each performed as six technical replicates; An unpaired Students *t* test was used to compare between two strains (PNAG).Two-tailed non parametric Mann-Whitney U test. (*P < 0.05; **P < 0.01; ***P < 0.001). IN, initial isolate; HA, host-adapted isolate; V_8_, the first isolate collected showing a SigB-deficient variant (isolate 15 after week 6).

We next investigated the capacity to form biofilms *in vitro*. The IN, HA and isolate 15 (V_8_) were grown statically at 37 °C for 24 hours in a 96-well plate, followed by crystal violet staining (Fig. 5A). The HA isolate formed significantly more biofilm than the IN isolate. Poly-*N*-acetylglucosamine (PNAG), the major polysaccharide component of the biofilm matrix, was quantified after staining with peroxidase-labelled wheat germ agglutinin (WGA) (Fig. 5B). Here, the HA isolate produced at least 70% more PNAG (P < 0.0001) compared to the IN isolate. Both SigB-deficient variants, V_8_ and V_9_ (HA), exhibited the same biofilm phenotype.

### Within-host adaptation towards attenuated virulence

**Figure 6:**
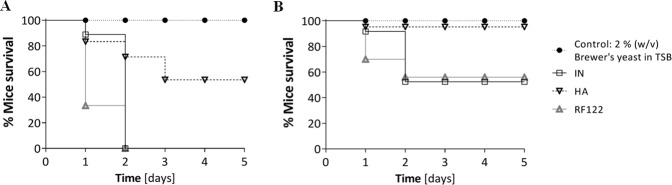
Bacterial virulence tested in an intraperitoneal murine infection model. Kaplan-Meier curves show the survival of mice after infection with either 1 × 10^7^ (**A**) or 1 × 10^6^ (**B**) CFU/ml bacterial inoculum, with five mice per group. Kaplan-Meier survival curves of the IN- and HA-infected mice were analyzed with the Log-rank test. IN, initial isolate; HA, host-adapted isolate.

An infection mouse model was used to investigate the difference in virulence between the mastitis isolates (Fig. 6). Female CF-1 outbred mice became moribund faster when infected with a higher (1 × 10^7^ CFU/mouse; Fig. 6A) than with a lower (1 × 10^6^ CFU/mouse; Fig. 6B) bacterial load, confirming a dose dependent virulence effect. For both doses, the IN isolate showed a faster progression and higher morbidity than the HA isolate. Almost no morbidity was observed (1 × 10^6^ CFU/mouse), when mice were challenged with the HA isolate. By contrast, RF122, a strain associated with severe mastitis, caused the fastest progression in morbidity, similar to the IN isolate.

## Discussion

In this work, we followed *S*. *aureus* within-host adaptation over the course of three months during a chronic, subclinical bovine mastitis by means of FTIR spectroscopic serotyping. Longitudinally collected *S*. *aureus* isolates shifted from encapsulation to non-encapsulation, an alteration associated with chronic infection^10,13^. Over the time course of sampling, the dairy cattle was not treated with any antibiotics. Thus, the fitness advantage of the within-host adapted *S*. *aureus* isolate reflects the response towards the bovine host.

As revealed by comparative whole-genome analysis, one dominant and several genetically heterogeneous subpopulations coexisted during the early phase of infection, differing by one or more additional single nucleotide mutations, which might have been effectively neutral or slightly detrimental. *S*. *aureus* mutation rates have been calculated to be equal to one SNP every 6 to 15 weeks^17^ and indeed, we did find 17 SNPs resulting in 9 clonal variants collected within 14 weeks. Within the timespan of our sampling, we observed the replacement of one dominant initial variant by another host-adapted variant, showing a SigB-deficient phenotype. The latter emerged equipped with a novel, stable trait profile from the first to the last isolate, which likely conferred a selective advantage as revealed by phenotyping.

To develop and maintain chronic infection, *S*. *aureus* within-host adaptation can lead to the emergence of specific pathotypes, such as small-colony (SCV) and *agr*-deficient variants^9,20^. In our present work, we were able to directly track the emergence of a SigB-deficient pathotype during the progression of a chronic, subclinical bovine mastitis. The SigB-deficient pathotype is likely caused by a missense mutation in *rsbU*, a gene that belongs to the *sigB* locus. One marker for SigB-activity, *asp23*^21^, was strongly down-regulated in the HA isolate at transcriptional and protein level. This was also seen for the *S*. *aureus* strain 8325-4 (11-bp deletion in *rsbu*) and SH1000∆*sigB* in comparison to SH1000 (fully functional SigB operon), supporting that the reduced *asp23* expression of HA can be attributed to SigB deficiency, which is associated to the detected SNP in the *rsbU* gene. Moreover, we observed a loss in pigmentation, reduced coagulase activity as well as an increase in proteolytic activity and Hla expression, all well-known indicators for SigB-deficiency^19,21^. SigB activity controls the expression of more than 200 genes and is regulated by a partner-switching mechanism in *S*. *aureus*^22,23^. One of the key points of this mechanism is the phosphorylation state of RsbV, where the phosphatase RsbU dephosphorylates RsbV for SigB activation. In contrast to *Bacillus subtilis*, RsbU in *S*. *aureus* might be constitutively active at high level, without any stimulation leading to an immanent SigB activation^24^. Since we detected the SNP in the *rsbU* gene (G368A) within the C-terminal phosphatase domain^24,25^, it is tempting to speculate that the resulting glycine/aspartate substitution (G122D) leads to a loss in the RsbV-P-specific phosphatase activity of RsbU, and thus sigB remains inactivated. Mutations affecting the SigB-functionality were previously detected *in vitro* (e.g. by consecutive passaging on culture media)^26,27^ as well as were consistently identified in isolates from human infections, such as the *S*. *aureus* laboratory reference strains NCTC8325 (11 bp deletion in *rsbU*) and KS26 (stop codon insertion in *rsbU*)^28,29^. Moreover, an adaptive evolution of *rsbU* was demonstrated during human chronic endobronchial infection of three cystic fibrosis patients, where a 18 bp in frame deletion was found in *rsbU*. Here, inferred mutation rates of the *sigB*-associated loci were 119-fold higher compared to the background mutation rate^30^. And just recently, a mutation in *rsbU* was described for *S*. *epidermidis* in-host evolution in a pacemaker-associated endocarditis^31^. Our data from a chronically infected dairy cattle as well as findings from human studies underscore the clinical relevance of the SigB-deficient pathotype in *S*. *aureus* pathogenesis, particularly in chronic, persistent infections.

Besides its central role in stress homeostasis, SigB contributes to several virulence determinants defining staphylococcal pathogenesis. This includes the transcriptional activation of a large number of surface proteins (such as *clfA*, *fnbpA*) while downregulating the production of secreted toxins and proteases (such as *aur*, *sspA*, *sspB*)^22^. Indeed, we found an exceptionally strong proteolytic activity in the SigB-deficient, host-adapted isolates linked to the mature exoprotease SspA, known to be the leading cause of staphylococcal proteolysis and part of a proteolytic cascade of activation. Aur, SspA and SspB are secreted as inactive zymogens and have to undergo a step-wise processing to be fully active. The Aur zymogen is autoactivated outside the cell, whereas activation of SspA and SspB relies on a proteolytic cascade in which Aur processes SspA and SspA subsequently processes SspB (Fig. 3D)^32,33^. Our data revealed a full activation of the proteolytic cascade for the HA in contrast to the IN isolate. This is experimentally supported by the strong transcriptional upregulation and subsequent secretion of Aur for the HA isolate and by the fact that the zymogens SspA and SspB remained in their inactive, proform in IN. Moreover, the differential abundance of several distinct SspA proteolytic cleavage products between the IN and HA isolate support that pro-SspA is converted to mature, active SspA solely in the HA isolate which is in in line with the step-wise, trypsinogen-like processing of SspA with the final activation step being critically dependent on Aur^18^. Increased proteolysis has been associated with persistence of *S*. *aureus* during chronic human osteomyelitis and cystic fibrosis^34,35^. It was suggested that the metabolism of persisting bacteria needs to adapt to a glucose-poor, but protein-rich environment of bone tissue, which may include the destruction of bone tissue^34^. Also degradation of host cell antimicrobial peptides and components of the complement system by *S*. *aureus* exoproteases was hypothesized^35^. Besides the direct influence on the host, staphylococcal exoproteases were shown to cleave bacterial surface proteins and are proposed to be important in the shedding of surface-associated proteins. In particular, staphylococcal fibronectin-binding proteins (FnBPs) are extremely susceptible to degradation by SspA^36^. In combination with a *sigB*-dependent expression of the *fnbpA* and *clfA* loci^22^, this could explain the diminished binding of the host-adapted, SigB-deficient isolate to immobilized fibronectin. High levels of secreted SspA and Aur have been reported to degrade Hla^37^, which possibly explains the reduced haemolytic activity on sheep-blood agar of the SigB-deficient, HA isolate, despite an elevated Hla expression and secretion^19^. Our data provide evidence that an enhanced proteolytic activity is advantageous in the course of a chronic infection, as it could possibly dampen bacterial immune recognition and toxicity to host cells, thus promoting access to new nutritional sources as well as escape from the host immune system, and might be responsible for the reduced virulence of the HA isolate in the intraperitoneal murine infection model.

Furthermore, internalization and survival of *S*. *aureus* in non-professional phagocytes represents an important mechanism in chronic, persistent infections^38^. It was shown in bovine mastitis, that *S*. *aureus* can invade and survive in mammary epithelial cells *in vitro* and viable *S*. *aureus* have been successfully isolated from alveolar cells, derived from milk of chronically infected cows^39–41^. Loss in capsule expression was associated with a higher internalization rate^35^, thus we expected that the non-encapsulated HA isolate internalize better. In contrast, we observed a slight, but significant decreased capacity to internalize into bovine epithelial cells, which is in line with previous findings, showing that activation of SigB is required for its cellular uptake^42,43^. Additionally, no differences were observed between the IN and HA isolate for intracellular survival up to 48 hours. Therefore, the HA, SigB-deficient phenotype seems less suited for the intracellular niche.

*S*. *aureus* biofilm formation has been linked to long-term persistence in bovine mastitis^44^. Indeed, we found higher levels of biofilm formation for the HA, SigB-deficient pathotype. We also observed an increase in the biofilm PNAG-content in the HA isolate, the main exopolysaccharide of the *S*. *aureus* biofilm matrix, produced via the *ica*-operon^45^. It is tempting to speculate that the increase in biofilm formation is primarily based on the PNAG-dependent exopolysaccharide rather than a proteinaceous biofilm matrix. Corroborating our findings, it has been shown recently that *S*. *aureus* sigB-deficient mutants produce higher PNAG-based biofilms compared to their corresponding wild-type strains, which was associated with a lower turnover of Ica proteins maintaining a higher rate of PNAG exopolysaccharide synthesis^46^. Therefore, the higher production of PNAG-based biofilms by the HA isolate might contribute to the capacity of strains to adapt to an extracellular niche facilitating staphylococcal persistence within the bovine host.

Given the significant phenotypic changes between the IN and HA isolate, alteration in *S*. *aureus* virulence could be expected. Accordingly, the HA, SigB-deficient isolate caused a significantly lower mortality in a mouse model of infection. The contribution of SigB to virulence is still under debate, since cases of both attenuated and retained virulence were observed^27,34^. It was proposed that a diminished SigB-functionality might not be detrimental^28^, and that the complex, fine-tuned regulatory SigB network rather is modulating virulence than determining it^47^. However, we found a clear trend towards reduced virulence of the HA isolate, as one would expect in chronic, persistent infections^48^.

In conclusion, we show that the SigB-deficient phenotype is of clinical relevance, particularly in chronic, persistent infections. We could demonstrate the emergence of a less virulent, SigB-deficient *S*. *aureus* pathotype as a consequence of bacterial adaptation to the infected bovine udder microenvironment. We identified proteolysis as an advantageous factor within the chronic infected mammary gland, possibly mediating immune evasion and exploitation of new nutritional sources. Moreover, the enhanced capacity to form PNAG-based biofilms possibly contributes to a better counteraction against immune attacks from the host and to a better adaption to the extracellular niche.

## Material And Methods

### Sampling, isolation and identification of bovine isolates

*S*. *aureus* isolates were recovered from milk samples derived from routine milking of a cow (Brown Swiss breed; manual milking stanchion and tethered housing at the University of Veterinary Medicine Vienna) with subclinical mastitis over a period of three months. During the study the animal did not show any symptoms of clinical mastitis. No bacteria were administered to the animal. Health management by a professional Veterinarian was independent from our study. Due to bronchitis with systemic administration of antibiotics, the milk sampling was discontinued after three months of sample collection. Aseptically collected milk samples were processed in frame of routine microbial mastitis diagnostics^8^.

### Reference strains and growth conditions

*S*. *aureus* bovine strain RF122 (ST151)^49^ was included as a reference strain shown to induce severe mastitis. Strain 6850^50^ and its isogenic ∆*sigB* mutant^51^ were used for phenotypic discrimination of coagulase and proteolytic activity as well as staphyloxanthin production. *S*. *aureus* 6850∆hla (provided by L. Tuchscherr de Hauschopp, Institute of Medical Microbiology, University Hospital of Jena, Germany), 6850^50^ and its isogenic ∆*sigB* mutant^51^ were used as control strains to test for the bovine isolates haemolytic activity on sheep blood agar. *S*. *aureus* SH1000^52^, 8325-4^53^ and SH1000∆SigB^43^ were included for mRNA expression studies of *asp23*. All *S*. *aureus* isolates were grown in tryptic soy broth (TSB) (Thermo Fisher Scientific, Oxoid, Hampshire, UK) supplemented with erythromycin (5 µg/ml) where needed.

### FTIR-based strain typing and CP serotyping

FTIR spectroscopic strain identification, subtyping and CP serotyping were performed as described previously^16,54^. Hierarchical cluster analysis (HCA)-assisted, high-resolution subspecies differentiation was performed at the spectral region for carbohydrate constituents (1,200–800 cm^−1^) using average spectra of measurements performed on three different days. CP serotypes (CP5, non-encapsulated) were determined by artificial neuronal network-assisted supervised chemometrics.

### Comparative whole-genome sequencing analysis

Isolates were sequenced using Illumina technology with 250-bp paired-end protocols on a MiSeq sequencer (Illumina). Libraries were adjusted to obtain a minimum of 100-fold sequencing coverage. Fastq files were trimmed (average base quality of 30, aiming for >100-fold coverage) and *de novo* assembled using Velvet 1.1.04 with SeqSphere + (version 5.1; Ridom, Münster, Germany)^55,56^. All genomes harbored ≥95% cgMLST targets and allele designations were assigned to the draft genome sequences provided by the SeqSphere software. In addition, Illumina paired-end reads of the initial (IN) and host-adapted (HA) isolate were trimmed using Trimmomatic version 0.38^57^. Draft genomes were *de novo* assembled with SPAdes version 13.13.0^58^, then annotated with Prokka version 1.13.3^59^.

### Staphyloxanthin production

One-milliliter of an overnight culture from each strain was precipitated by centrifugation (10,000 g for 1 minute) after the optical density (OD) at 600 nm was measured. The cells harvested were suspended in 200 µl of methanol and incubated at 55 °C for 3 minutes. After the cell debris was removed by centrifugation, the methanol extraction step was repeated once and the extracts were collected in the same tube. The pigment extracted was quantified by evaluation of the OD at 450 nm and normalized with the culture OD 600 nm^60^.

### Coagulase, proteolytic and hemolytic activity

Slide and tube agglutination were tested using rabbit plasma according to the manufacturer’s instructions, Thermo Scientific™ Remel, #R21060. Strains were tested for their proteolytic capacity, by growing them on either 1% Skim milk agar or in 1% Skim milk broth, for 18 hours at 37 °C. Proteolytic activity was assessed by eye and compared to the growth medium without any bacterial load. Haemolytic activity was tested by spotting 5 µl of overnight culture (TSB) onto CBA and direct haemolysis evaluation after 18 hours at 37 °C, followed by a further 4 hour incubation at 4 °C, to test for “cold haemolysis“.

### Zymography

Zymography was performed according to Bose *et al*.^61^. Briefly, the reduced samples (7.5 to 15 µg protein/lane) were loaded onto a 12.5% SDS-PAGE gels supplemented with 1% casein. Proteins were separated at 120 Volts, with a maximum of 20 mA at 4 °C. After regeneration in 2.5% Triton X-100 and successive equilibration with 5 mM CaCl_2_, 50 mM Tris at pH 7.4 for 20 minutes and 0.2 M NaCl, 10 mM CaCl_2_, 50 mM Tris, 0.02% Brij35 at pH 7.4 for 20–30 hours, the gel was stained overnight at room temperature using PageBlue Protein staining and destained with ultrapure water.

### 2D-DIGE and MALDI-TOF MS/MS

Two-dimensional difference gel electrophoresis (2D-DIGE) and MALDI-TOF MS/MS-based protein identification was carried out as previously described^62^. Briefly, CyDye DIGE^TM^ fluorescent dyes (GE Healthcare LifeScience) were used to label proteins of bacterial supernatants obtained by precipitation in 10% TCA. Isoelectric focusing was conducted on an IPGphor III system using cuploading on 24 cm IPG Dry strips/ pH 3-10NL (all GE Healthcare LifeScience). The second dimension was conducted in an Ettan DALTsix electrophoresis system (GE, Healthcare). Fluorescence images of the 2D-DIGE gels were acquired on a Typhoon 9400 scanner (GE Healthcare) and images were edited using ImageQuant TL software version 8.1 (GE Healthcare).

Protein spots were visualized by MS-compatible silver staining, manually excised, pooled and destained as previously described^63^. Digestion was carried out for 8 hours at 37 °C using 12.5 μg/μl trypsin (Promega). Extracted peptides were concentrated and desalted using μZipTips C18 (Millipore). Proteins were identified by means of Matrix Assisted Laser Desorption Ionization Tandem Time-of-Flight (MALDI-TOF/TOF) mass spectrometer (Ultraflex II, Bruker Daltonics, Germany) using pre-spotted with α-cyano-4-hydroxycinnamic acid (PAC target, Bruker Daltonics). Spectral preprocessing and peak annotation was carried out using FlexAnalysis and Biotools (Bruker Daltonics). Protein lists were searched at UniProt limiting the taxonomy to *S*. *aureus* strain COL and USA300, using the following parameters: MS tolerance 100 ppm; MS/MS tolerance 1 Da; one missed cleavage allowed; fixed modifications: carbamidomethylation of cysteins and variable modifications: Oxidation (M), Deamidated (NQ), Gln- > pyro-Glu (N-term Q). Identifications were considered statistically significant for proteins if Mascot >80.0 and for peptides if Mascot >20.0.

### Western Blotting

Western blots were performed on small-sized SDS-PAGE gels as described previously^64^. Monoclonal anti-Hla-Ab (#8950), kindly provided by Arsanis Biosciences (Vienna, Austria) was used as primary antibody, followed by the secondary antibody goat F(ab′)2 Anti-Human lgG (H + L)-HRP (Southern Biotech, Birmingham, AL, USA) and detection by ECL Western Blotting Detection reagents (GE Healthcare Life Sciences).

### Cell culture and internalization and survival assay

For all cell-culture experiments, a bovine mammary epithelial cell (MAC-T) line was used^65^. Internalization and survival experiments were performed similar to Buzzola *et al*.^66^. To detect bacterial survival at 24 and 48 hours post infection, initial lysostaphin treatment after 1 hour of cell-bacteria co-incubation, was repeated with fresh medium every 24 hours, assuring the complete removal of extracellular staphylococci^67^. Experiments were performed at least three times in technical triplicates.

### Fibronectin binding

*S*. *aureus* binding to immobilized bovine fibronectin was examined similar to the protocols by Brouillette *et al*.^68^ and Marbach *et al*.^69^. Aliquotes of exponential phase cultures (1 × 10^8^ CFU) were tested for binding to 5 mg/ml bovine fibronectin (Sigma-Aldrich). Bound bacteria were stained with 0.1% crystal violet, washed with Milli-Q water, the plates dried and fibronectin binding quantified by measurement at an OD of 590 nm (SpectraMax M5 Absorbance Microplate Reader, Molecular Devices) after dissolving bacteria bound crystal violet in 30% (v/v) glacial acetic acid. Experiments were performed at least three times, each with six technical triplicates.

### Biofilm formation and PNAG production

Quantitative biofilm and semi-quantitative PNAG assessment was performed as described earlier^8^. Results were evaluated from three independent experiments, each performed with six technical replicates.

### Real-time PCR

RNA extraction and qRT-PCR was performed according to the protocol described in Dommel *et al*.^5,70^. Total RNA extraction was performed on bacterial strains grown at 37 °C in TSB for zero (OD600 0.05), 3 and 6 hours (120 rpm and aerobe). Primers and their efficiencies are listed in Table S2. Following cycling conditions on the on the C1000 Touch Thermal Cycler CFX96 Real-Time System (BioRad) were used: polymerase activation and DNA denaturation at 95 °C/3 minutes, followed by 35 cycles of denaturation at 95 °C/10 seconds and annealing/extension at 54 °C (*asp23*)^43^, 59 °C (*aur*)^43^, 59 °C (*hla*)^71^, 59.1 °C (*rho*), 54.5 °C (*rpoD*) and 59.2 °C (*dnaN*)^42^ for 30 seconds, followed by a melting curve analysis between 65–95 °C in steps of 0.5 °C/5 seconds. Relative expression levels were calculated using the REST method by Pfaffl, with internal calibration to samples retrieved at time point zero and normalized to the geometric mean of three reference genes (*rpoD*, *rho* and *dnaN*). Mean relative expression and standard deviations were calculated from three independently grown samples and their technical duplicates.

### Lethal infection murine model

Female CF-1 outbred mice were bred and maintained in the vivarium the Instituto de Investigaciones en Microbiología y Parasitología Médica (Universidad de Buenos Aires-CONICET). This study was carried out in accordance with the recommendations of the international guidelines set forth by: the 11 report of the BVAAWF/FRAME/RSPCA/UFAW Joint Working Group on Refinement. The protocol was approved by the Institutional Animal Care and Use Committee (CICUAL), resolution N2780/2018 of the School of Medicine, University of Buenos Aires.

Groups of 5 mice at an age of 2 months were infected by intraperitoneal (i.p.) injection with 0.5 ml of a suspension containing 1 × 10^6^ or 1 × 10^7^ CFU of *S*. *aureus* IN, HA or RF122 strains and 2% (w/v) Brewer’s yeast (Sigma Chemical Co.) in TSB broth^72^. The number of CFU in the inoculum was verified by viable counts after plating an aliquot of serial dilutions on TSA plates. The control group was injected with 0.5 ml of 2% (w/v) Brewer’s yeast in TSB broth by i.p. route. Mice were monitored daily until day five. The criteria to establish that the endpoint (systemic infection/morbidity) was reached were the following: i) loss of at least 15% of body weight with or without ruffled or “spikey” fur and ii) loss of at least 10% of body weight and hunched posture^73^. Animals that presented these signs were sacrificed and were scored as dead in the survival analysis. Mice were euthanized by CO_2_ inhalation and subsequent cervical dislocation.

### Statistics

The difference between two strains was tested by an unpaired Students *t*-test (two-tailed), unless the data was identified as not normally distributed. Under these circumstances, the non-parametric Mann-Whitney U test (two-tailed) was used. Minimum statistical significance was set to P < 0.05. For the survival data, Kaplan-Meier survival curves were plotted and analyzed using the Log-rank test. The GraphPad Prism7.0 software was used for all statistical calculations.

## Supplementary information

Supplementrary Info

## Data Availability

The datasets generated and/or analyzed during the current study are available from the corresponding author on request.
