# Supplementary material for: Within-host evolution of bovine Staphylococcus aureus selects for a SigB-deficient pathotype characterized by reduced virulence but enhanced proteolytic activity and biofilm formation
Source: Sci Rep. 2019 Sep 17;9:13479. doi: 10.1038/s41598-019-49981-6 (PMC6748969; doi:10.1038/s41598-019-49981-6)
Supplement: Supplementary file 1 — Supplementrary Info [file 41598_2019_49981_MOESM1_ESM.pdf]

Supplementary Information

Within-host evolution of bovine *Staphylococcus aureus* selects for a SigB-deficient pathotype characterized by reduced virulence but enhanced proteolytic activity and biofilm formation

Helene Marbach[1], Katharina Mayer[1], Claus Vogl[2], Jean Y.H. Lee[3], Ian R. Monk[3], Daniel O. Sordelli[4], Fernanda R. Buzzola[4], Monika Ehling-Schulz[1], Tom Grunert[1,*]

[1] Functional Microbiology, Institute of Microbiology, Department of Pathobiology, University of Veterinary Medicine, Vienna, Austria.

[2] Molecular Genetics, Institute of Animal Breeding and Genetics, Department of Biomedical Sciences, University of Veterinary Medicine, Vienna, Austria.

[3] Department of Microbiology and Immunology, Peter Doherty Institute for Infection and Immunity, University of Melbourne, Melbourne, Australia.

[4] Instituto de Investigaciones en Microbiología y Parasitología Médica (IMPaM), Universidad de Buenos Aires and CONICET, Buenos Aires, Argentina.

Figure S1. (A) Staphyloxanthin production **(B)** Tube coagulase **(C)** Slide agglutination test.

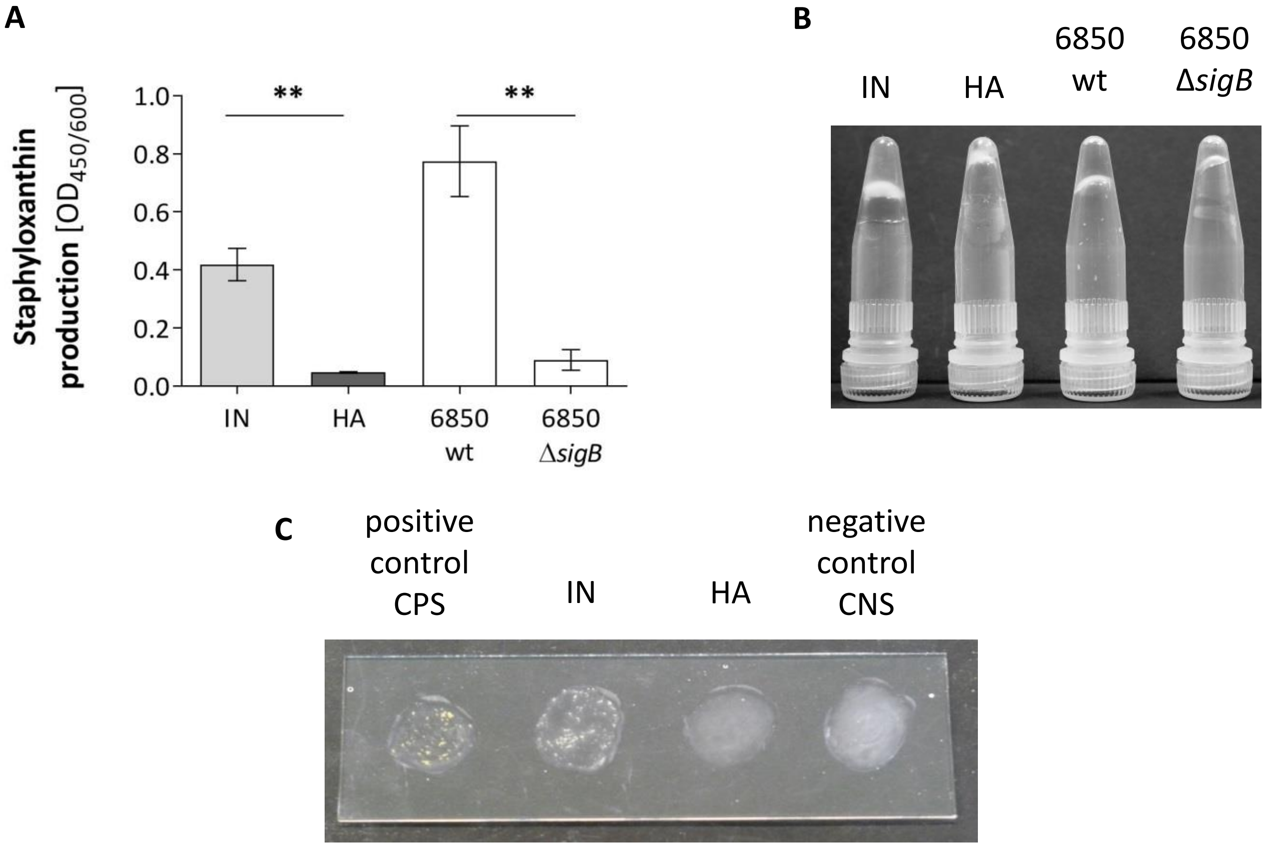

Difference in colony pigmentation intensity is shown as the mean ± SEM of three independent experiments with samples measured in duplicate, and statistical difference between the isolates calculated, using the unpaired Students *t* test. (**, $P<0.01$); CPS-coagulase positive staphylococci, CNS-coagulase negative staphylococci; IN, initial isolate; HA, host-adapted isolate.

Figure S2. *asp23* RT-qPCR

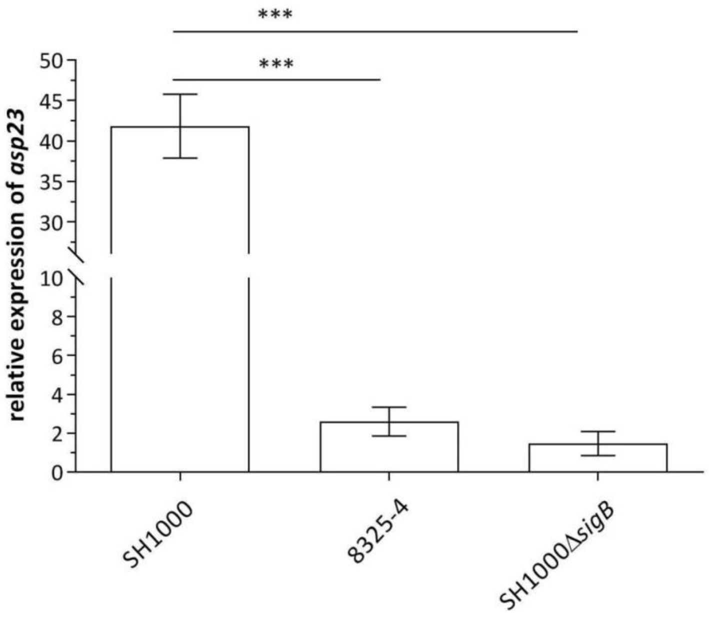

Asp23 expression of strains grown in TSB to an OD 600 nm of 2.5 was evaluated by RT-qPCR. The data shown are the means of three independently grown strains and their technical duplicates. Statistical difference between the expression of two isolates was determined with unpaired Students *t* test on log10 transformed data. (***, P<0.001).

Figure S3. Proteolytic activity: Milk proteolysis on **(A)** 1% Skim milk agar and **(B)** 1% Skim milk (IN, initial isolate; HA, host-adapted isolate).

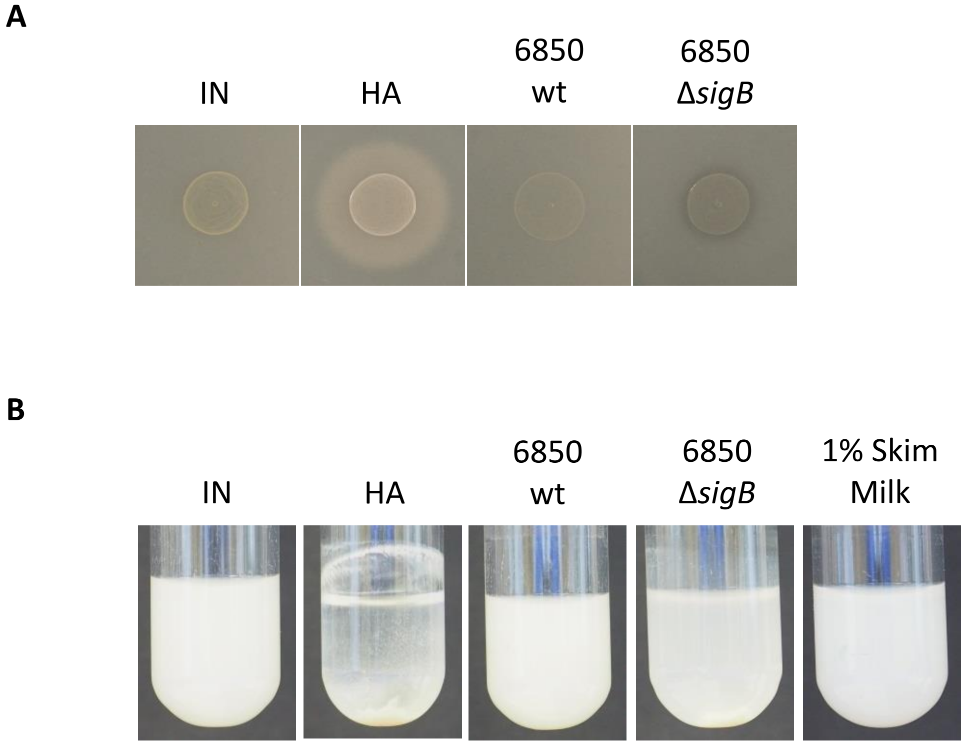

Figure S4. MALDI-TOF-MS/MS-based protein identification.

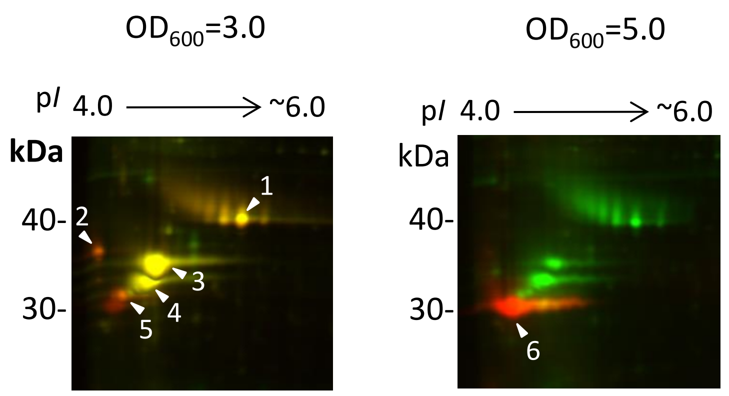

Spot No.	Gene name	Uni-Prot Accession No.	Search results of Uni-Prot against *S. aureus* strain COL and alternative name	MW (kDa) theor.	pI theor.	Probability based MASCOT score	Sequ. Cov. %	Matched peptides MS/MS	Range	m/z measured	Peptide score	Peptide sequence [#]
1	**SspB**	Q5HH36	**Staphopain B** OS=Staphylococcus aureus (strain COL) OX=93062 **GN=sspB** PE=3 SV=1; **alternative name: cysteine protease**	44.5	5.6	503.8	23.9	6	56 - 69	1566.7682	71.4	K.VKDLAQQQFAGYAK.A
									93 - 107	1837.7629	82.4	K.FNGEEDNSYYYPVIK.D
									153 - 174	2561.1206	106.9	K.NSNITVLTDEKGFYFEEDGKVR.L
									164 - 174	1346.5929	71.7	K.GFYFEEDGKVR.L
									299 - 317	2248.9729	90.3	R.DIHYQEGVPSYEQVDQLTK.D
									233 - 256	2758.2146	81.1	K.IREQQFDNSWCAGFSMAALLNATK.N
2	**Aur**	A0A0H2WZZ4	**Zinc metalloproteinase aureolysin** OS=Staphylococcus aureus (strain COL) OX=93062 **GN=aur** PE=4 SV=1; **alternative name: Aureolysin**	56.3	5	275.1	16.5	3	245 - 273	3318.7454	128.9	K.LSAFSFNDQTGQATLITNEDENFVKDEQR.A
									296 - 320	2765.3381	104.3	R.ESYDNQGSPIVSLTHVNNYGGQDNR.N
									339 - 368	3234.6721	41.8	R.TFTSLSGANDVVAHELTHGVTQETANLEYK.D
3	**SspA**	Q5HH35	**Glutamyl endopeptidase** OS=Staphylococcus aureus (strain COL) OX=93062 **GN=sspA** PE=3 SV=1; **alternative names: Staphylococcal serine protease A, Endoproteinase Glu-C, V8 protease**	36.3	4.9	482.4	21.1	4	55 - 64	1111.7092	33.1	K.GGNLKPLEQR.E
									65 - 76	1391.8103	43.2	R.EHANVILPNNDR.H
									133 - 155	2484.2935	198.6	K.AFPSAINQDNYPNGGFTAEQITK.Y
									246 - 271	2882.5601	207.5	K.NEVIGIHWGGVPNEFNGAVFINENVR.N
4	**SspA**	Q5HH35	**Glutamyl endopeptidase** OS=Staphylococcus aureus (strain COL) OX=93062 **GN=sspA** PE=3 SV=1; **alternative names: Staphylococcal serine protease A, Endoproteinase Glu-C, V8 protease**	36.3	4.9	460.9	17.6	4	55 - 64	1111.6447	86	K.GGNLKPLEQR.E
									65 - 76	1391.7267	82.2	R.EHANVILPNNDR.H
									119 - 132	1496.7985	122.8	K.HVVDATHGDPHALK.A
									133 - 155	2484.2112	169.8	K.AFPSAINQDNYPNGGFTAEQITK.Y
5	**SspA**	Q5HH35	**Glutamyl endopeptidase** OS=Staphylococcus aureus (strain COL) OX=93062 **GN=sspA** PE=3 SV=1; **alternative names: Staphylococcal serine protease A, Endoproteinase Glu-C, V8 protease**	36.3	4.9	347.7	28.6	4	65 - 76	1391.7535	63.7	R.EHANVILPNNDR.H
									77 - 111	3672.9709	96.1	R.HQITDTTNGHYAPVTYIQVEAPTGTFIASGVVVGK.D
									133 - 155	2484.3547	127.3	K.AFPSAINQDNYPNGGFTAEQITK.Y
									246 - 271	2881.5850	60.5	K.NEVIGIHWGGVPNEFNGAVFINENVR.N
6	**SspA**	Q5HH35	**Glutamyl endopeptidase** OS=Staphylococcus aureus (strain COL) OX=93062 **GN=sspA** PE=3 SV=1; **alternative names: Staphylococcal serine protease A, Endoproteinase Glu-C, V8 protease**	36.3	4.9	376.2	25.6	4	119 - 132	1496.7922	50.3	K.HVVDATHGDPHALK.A
									133 - 155	2484.3079	130.2	K.AFPSAINQDNYPNGGFTAEQITK.Y
									223 - 245	2390.1846	129.4	K.GEAMQYDLSTTGGNSGSPVFNEK.N
									246 - 271	2882.4939	66.2	K.NEVIGIHWGGVPNEFNGAVFINENVR.N

[#] MS/MS-sequence tags of tryptic peptides derived from the protein's propeptide were highlighted in blue (see also Figure S5).

Figure S5. Conversion of pro-SspA to mature, active SspA in HA

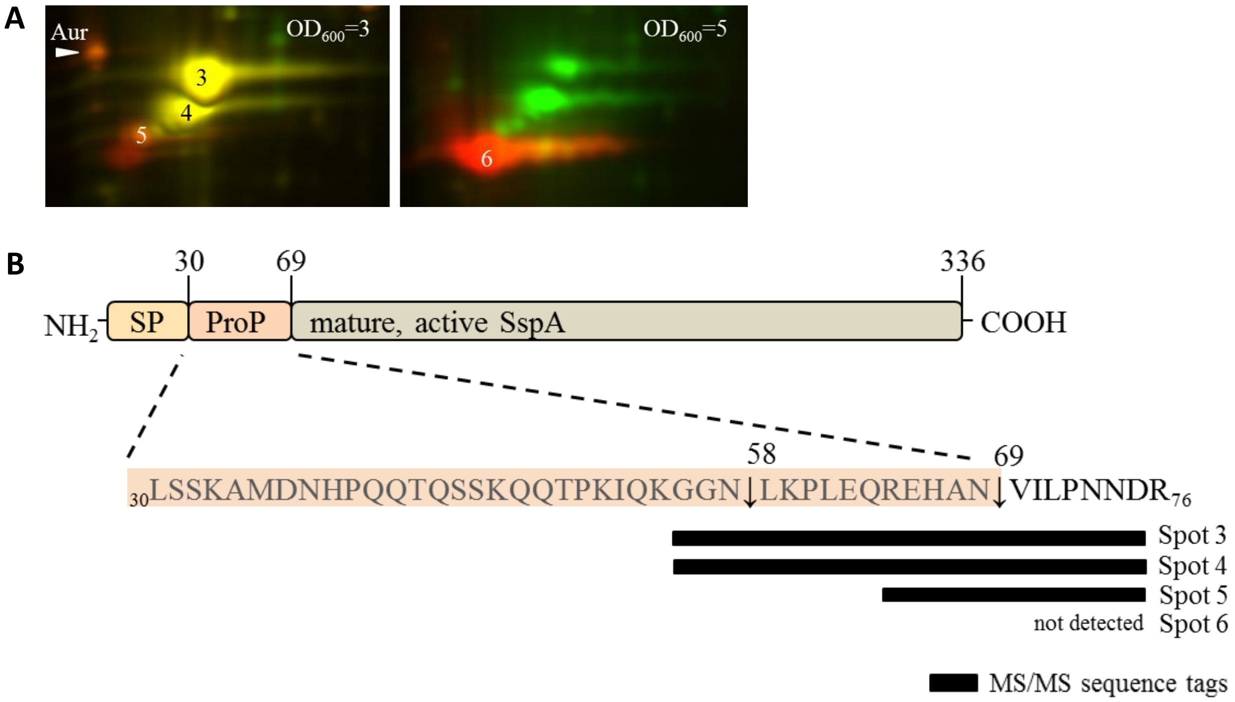

The trypsinogen-like mechanism of SspA activation was described by Nickerson *et al.* 2007, J Biol Chem.23;282(47):34129-38. Nickerson *et. al.* 2007 demonstrated that after initial processing through autocatalytic intramolecular cleavage within a glutamine-rich SspA-propeptide segment, ^{40}QQTQSSKQQTPKIQ^{53}, Aur subsequently processes at Leu^{58} and then Val^{69} to release mature, active SspA.

Indeed, for the SspA subform with the lowest molecular weight (Fig. S5A; spot 6) which was only abundant in the supernatant of HA (red channel), no peptide segments derived from the propeptide (pro-SspA) were detected by MALDI-TOF-MS/MS (Fig. S5B; see also Fig. S4). In contrast, all analysed higher molecular weight subforms of SspA (S5A; spots 3-5) contained peptide segments derived from the propeptide (Fig. S5B) which can be associated to SspA intermediates as a result of it's step-wise activation being critically dependent on Aur at the final activation step. In line, Aur was only detected for the HA, but not in the IN isolates' supernatant at protein level which was supported by a very strong upregulation of *aur* at the transcriptional level in HA (Fig. 3C).

Thus, the differential abundance of SspA proteolytic cleavage products between the IN and HA isolates as well as specifically, detected MS/MS sequence tags derived from the propeptide of SspA support that pro-SspA is converted to mature, active SspA solely in the HA strain which further results in the subsequent activation of the zymogen SspB by removing its N-terminal propeptide as demonstrated by Massimi et al. 2002, J. Biol. Chem. 277, 41770–41777. Consequently, pro-SspB could not be detected in HA, but was present in the IN supernatant at OD_{600} of 5.0 (Fig. 3b; Fig. S4).

Figure S6. (A) relative *hla* expression **(B)** Hla protein secretion **(C-D)** Haemolytic activity; after 18 hours at 37°C **(C)** and further storage at 4°C for 4 hours **(D)** plate layout **(E)**.

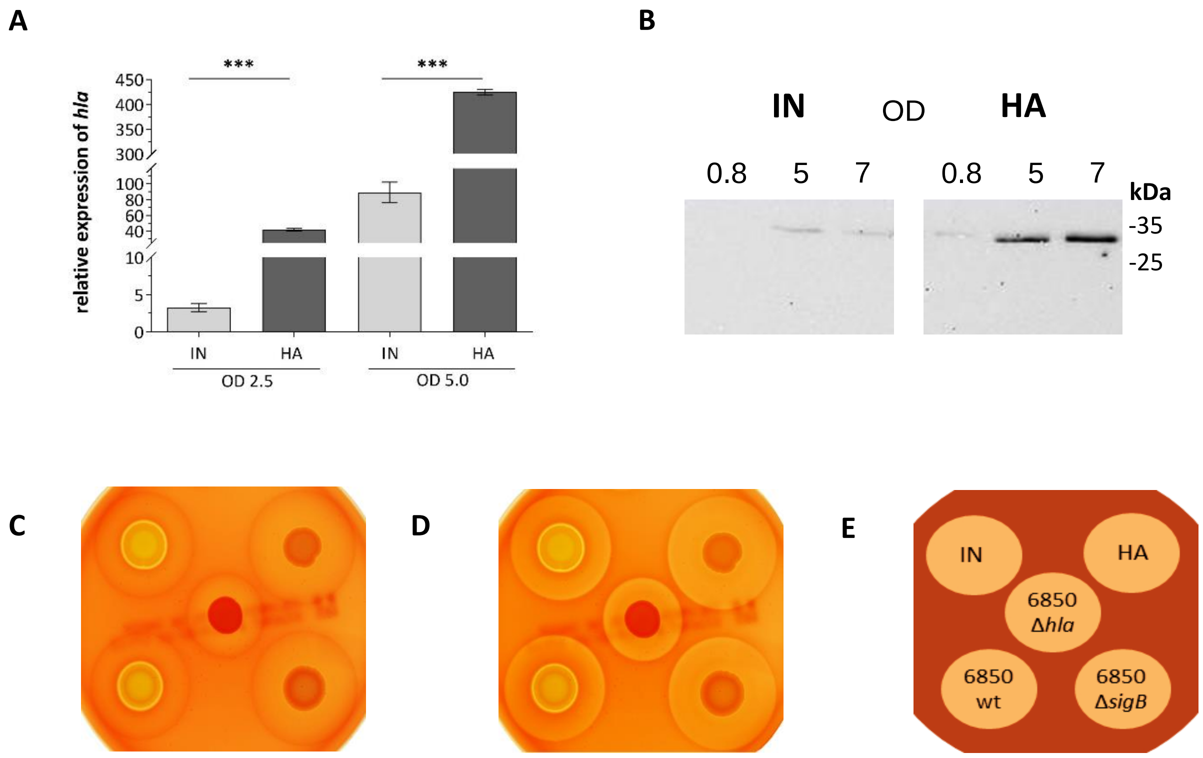

(A) *Hla* expression (qRT-PCR) was performed on strains grown in TSB to an optical density of 2.5 and 5.0. The data shown are the means of three independently grown strains and their technical duplicates. Statistical difference between the expression of the initial and host-adapted isolate was determined with unpaired Students *t* test on log10 transformed data. (***, P<0.001).

(B) SDS-Page and western blot was performed with bacterial supernatant of three different bacterial densities (OD_{600} = 0.8, 5 and 7) against the monoclonal anti-Hla-antibody.

(C-E) Haemolytic activity was assessed by spotting bacteria onto sheep blood and incubation. Zone of blood clearance was read by eye.

IN, initial isolate; HA, host-adapted isolate.

Figure S7. Original, full length zymogel/ 2D-DIGE gels for Figure 3A and 3B

3A

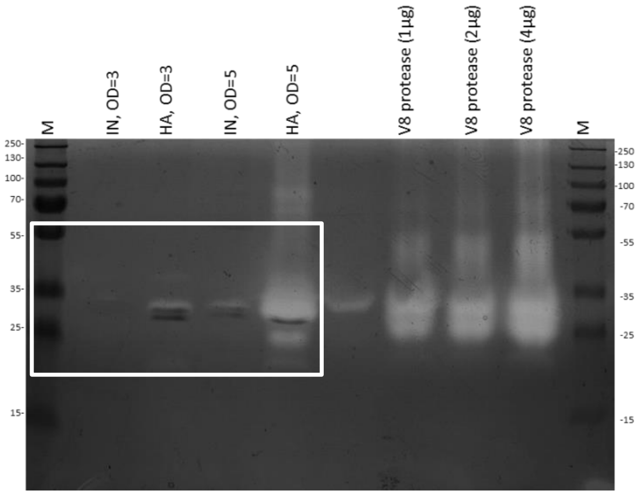

3B

OD_{600}=0.8

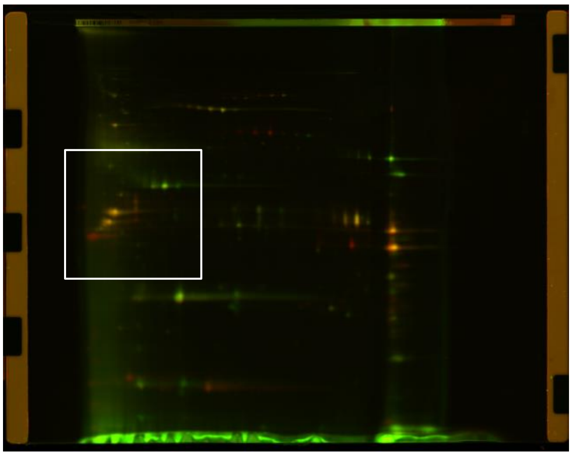

OD_{600}=3.0

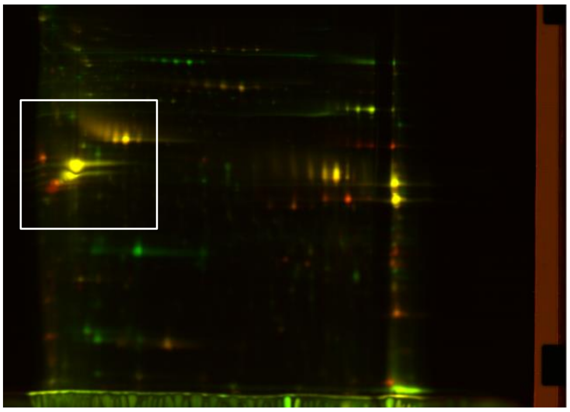

OD_{600}=5.0

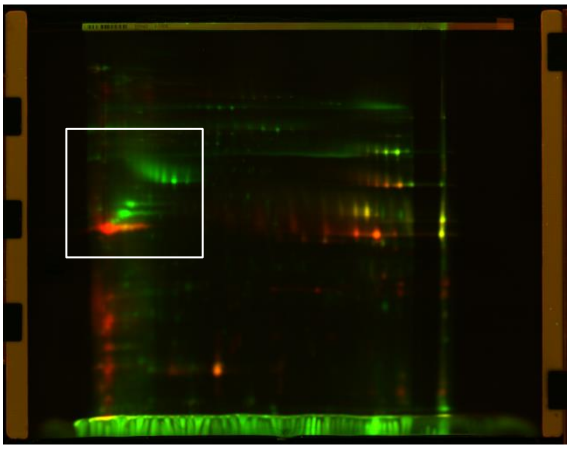

Table S1. Mutations (SNPs) detected by comparative sequence analysis of the initial isolate (V1) and its isogenic isolates.

Variant	Locus tag (N315)	Locus tag (COL)	Gene	Product	Effect	Function
V2	SA1026	SACOL1196	murD	UDP-N-acetylmuramoyl-L-alanyl-D-glutamate synthetase	non-synonymous	Biosynthesis and degradation of murein sacculus and peptidoglycan
V3	SA1891	SACOL2079	cls2	Cardiolipin synthetase	non-synonymous	Glycerolipid and Glycerophospholipid Metabolism
V4	SA0793	SACOL0935	dltA	D-alanine-poly(phosphoribitol) ligase subunit 1	non-synonymous	Biosynthesis and degradation of murein sacculus and peptidoglycan
	SA0834	SACOL0978	?	Hypothetical protein	non-synonymous	Unknown
	SA2129	SACOL2332	galM	Aldose 1-epimerase	non-synonymous	Lactose and Galactose Uptake and Utilization
	SA0315	SACOL0397	sirTM	Hypothetical protein	synonymous	Unknown
V5	SA1088	SACOL1262	sucC	Succinyl-CoA synthetase subunit beta	non-synonymous	TCA cycle; catalyzes the interconversion of succinyl-CoA and succinate
	SA0981	SACOL1144	isdF	Hypothetical protein	synonymous	Putative ABC transporter
V6	SA2147	SACOL2353	tcaR	Transcriptional regulator TcaR	non-synonymous	Quorum sensing and biofilm formation
	SA1675	SACOL1916	?	Amino acid ABC transporter permease/substrate-binding prote	synonymous	Putative ABC transporter
V7	SA2135	SACOL2340	gltS	Sodium/glutamate symporter	non-synonymous	Sodium/glutamate symporter
	SA1990	SACOL2179	?	Hypothetical protein	synonymous	Unknown
	SA2084	SACOL2282	ureC	Urease subunit alpha	synonymous	Urea decomposition
V8	SA1872	SACOL2057	rsbu	Serine phosphatase RsbU, regulator of sigma subunit	non-synonymous	SigmaB stress responce regulation
	SA0212	SACOL0198	?	Hypothetical protein	non-synonymous	Unknown
	SA0192	N.N.	?	Hypothetical protein	non-synonymous	Putative export ABC transporter
V9	SA1872	SACOL2057	rsbu	Serine phosphatase RsbU, regulator of sigma subunit	non-synonymous	SigmaB stress responce regulation
	SA0212	SACOL0198	?	Hypothetical protein	non-synonymous	Unknown
	SA0192	N.N.	?	Hypothetical protein	non-synonymous	Putative export ABC transporter
	SA1828	N.N.	?	Hypothetical protein	non-synonymous	Putative DNA helicase

Whole genome sequencing was performed by shotgun sequencing using the *Illumina MiSeq* platform PE300. SNP differences of 20 isolates compared to the IN isolate (V1), allowing for gene variant assignment based on the strains SNP difference. Locus tags referred to the *Staphylococcus aureus* strains N315 and COL.

Table S2. qRT-PCR primer and their efficiencies.

Target gene	Forward primer (5' – 3')	Reverse primer (5' – 3')	Primer efficiency	Reference
dnaN	ACGTGATTGCACAAACAAATTTTGC	AAGCCAGTTCACACCAGTTAGTAC	2.38	Pförtner *et al*., 2014, *Int. J. Med. Microbiol.* 304, 177–187.
rho	CAGGACGCTCGCCAACTAAC	CCGCATTTCCAAGCTTTGACAC	2.00	current study
rpoD	TCAAGGAATAACATACCACGAC	TGACGAGAAACTGAATCCAAG	1.91	current study
hla	GCAAATGTTTCGATTGGTCA	CCATATACCGGGTTCCAAGA	2.05	Burnside *et al.,* 2010, PLoS One 5:e11071
asp23	AAAGCAAAACAAGCATACGACAATC	AGCGATACCAGCAATTTTTTCAAC	1.99	Tuchscherr *et al.*, 2015, *PLoS Pathog.* 11, e1004870.
aur	ACCGTGTGTTAATTCGTGTGCTA	ATGGTCGCACATTCACAAGTTT	2.07	Tuchscherr *et al.*, 2015, *PLoS Pathog.* 11, e1004870.
